# Development of memory clinics in the Netherlands over the last 20 years

**DOI:** 10.1002/gps.5132

**Published:** 2019-05-20

**Authors:** Angélique A.A. Gruters, Inez H.G.B. Ramakers, Roy P.C. Kessels, Femke H. Bouwman, Marcel G.M. Olde Rikkert, Marco M. Blom, Marjolein E. de Vugt, Frans R.J. Verhey

**Affiliations:** ^1^ Department of Psychiatry and Neuropsychology, School for Mental Health and Neuroscience, Alzheimer Center Limburg Maastricht University Maastricht The Netherlands; ^2^ Donders Institute for Brain, Cognition and Behaviour Radboud University Nijmegen Nijmegen The Netherlands; ^3^ Department of Medical Psychology and Radboudumc Alzheimer Center Radboud University Medical Center Nijmegen The Netherlands; ^4^ Alzheimer Center and Department of Neurology VU University Medical Center, Amsterdam Neuroscience Amsterdam The Netherlands; ^5^ Radboudumc Alzheimer Center and Department of Geriatrics Radboud University Medical Center Nijmegen The Netherlands; ^6^ Alzheimer Nederland Amersfoort The Netherlands

**Keywords:** diagnosis and classification, dementia, memory clinics, services, mild cognitive impairment (MCI)

## Abstract

**Objectives:**

Memory clinics (MCs) have been established to improve diagnosis and treatment of cognitive disorders, including dementia. The aim of this study was to determine the characteristics and working methods of MCs in the Netherlands in 2016. More insight into different working methods can be used to improve the quality of care in Dutch MCs. Additionally, the findings will be compared with earlier results to investigate the development of MCs since 1998.

**Methods:**

A survey was sent in 1998, 2004, 2009, and 2017 to all operational Dutch MCs with questions about organization, collaboration, patients, and diagnostic procedures.

**Results:**

From 1998 to 2016, the number of MCs increased substantially from 12 to 91. The capacity increased from 1560 patients to 24,388. In 1998, most patients received a dementia diagnosis (85%), while in 2016, half of the patients were diagnosed with milder cognitive problems. MCs are more often part of regional care chains and are better embedded within regional care organizations. Diagnostic tools, such as blood tests (97%), neuropsychological assessment (NPA) (95%), and neuroimaging (92%), were used in nearly all MCs. The number of patients in whom these tools were used differed greatly between MCs (NPA: 5%‐100%, neuroimaging: 10%‐100%, and CSF: 0.5%‐80%). There was an increase in the use of NPA, while the use of neuroimaging, CSF, and EEG/ECG decreased by 8% to 15% since 2009.

**Conclusions:**

Since 1998, MCs have developed substantially and outgrown the primarily research‐based university settings. They are now accepted as regular care facilities for people with cognitive problems.

**Key points**

- Memory clinics in the Netherlands developed considerably in the last 20 years and are now accepted as regular care facilities for people living with cognitive problems and dementia.
- People with cognitive problems attend the memory clinic in an earlier disease stage.
- Memory clinics in the Netherlands are increasingly collaborating with other regional care facilities and have psychosocial interventions more often as part of their treatment options.

## 1 INTRODUCTION

Early diagnosis of dementia enables improved understanding of the disease process, provides the opportunity to make decisions concerning the future while cognitive capacities are still relatively intact,^1^ and creates a time window to institute early interventions and support for patients and caregivers.^2^, ^3^ With no cure available, early diagnosis may also have some drawbacks. An increasingly widespread view acknowledges “timely diagnosis” as a more appropriate concept because it emphasizes a person‐centered approach in which the diagnosis is related to the benefit of the patient and not to a disease stage.^4^, ^5^ To improve early yet timely diagnosis, the development of specialized multidisciplinary memory clinics (MCs) has been promoted and recommended by national dementia strategies.^6^, ^7^, ^8^, ^9^, ^10^, ^11^, ^12^ The first clinics were established during the 1970s in the USA. During this time period, the perspective on the cause of dementia shifted from being an inevitable result of aging to being a disease.^13^ The past three decades have shown significant growth in the development of MCs worldwide. The increasing number of MCs has been explained by the increasing prevalence of dementia, licensing of pharmacological treatments, and the improvement in care services.^14^, ^15^, ^16^ A comparable increase in MCs can be seen in the Netherlands. To gain more insight into the development and efficacy of these clinics, a first national survey in the Netherlands, the MC Monitor, was published in 1998.^17^ At that time, the authors described MCs as often being established in university‐based hospitals with a focus on scientific research. The MC Monitor was repeated in 2004 and 2009 and showed an increase in the number and in the capacity of MCs.^18^, ^19^ Since 2009, new MCs have been established, and the Dutch guideline *Diagnostics and Treatment of Dementia* was revised in 2014.^20^ The aim of this study was to determine the characteristics of MCs in the Netherlands in 2016. Two key topics of the survey were neuropsychological assessment and regional collaboration. More insight into different working methods can be used to improve the quality of care in MCs. In addition, the results were compared with the findings of the previous surveys to investigate the development of MCs in the last 20 years.

## 2 METHODS

To gain more insight into the characteristics of MCs, a semistructured questionnaire was sent out in 1998, 2004, 2009, and 2017 to all hospital‐based MCs in the Netherlands asking for data from the previous year. An MC was defined as a multidisciplinary team with at least two disciplines (at least one medical profession) dedicated to the diagnosis of dementia. All relevant operational clinics were identified using the network of the Alzheimer Center Limburg and through internet searches. Every survey consisted of core items that were repeated in each survey. In addition, relevant items were added over time by an expert group. In addition, the survey was piloted in three academic hospital‐based Dutch Alzheimer Centers. In this survey, the following topics were included: organization, collaboration, number of patients, distribution of diagnosis and etiology, referrals, procedures, diagnostic criteria, additional assessments, neuropsychological assessment, treatment, policy, and professionalization. Participants were asked to answer questions by using information derived from official sources, but if this was unavailable, they were allowed to use estimations. In 1998 and 2004, the survey was sent by mail and then by e‐mail and mail in 2009. In 2017, the survey was digitalized using Qualtrics software. MCs that did not respond received multiple reminders. A total of 91 MCs were identified, 78 of which returned the survey, resulting in a response rate of 86%. This was comparable with the previous surveys in 1998 (88%), 2004 (93%), and 2009 (78%). Most respondents were medical doctors. Statistical analyses were conducted using version 24 of the Statistical Package for Social Sciences (SPSS). To examine group differences between coordinating disciplines and university versus non‐university‐based MCs, analysis of variance (ANOVA) or independent *t‐*tests were conducted.

## 3 RESULTS

### 3.1 Number and capacity of MCs

**Figure 1:**
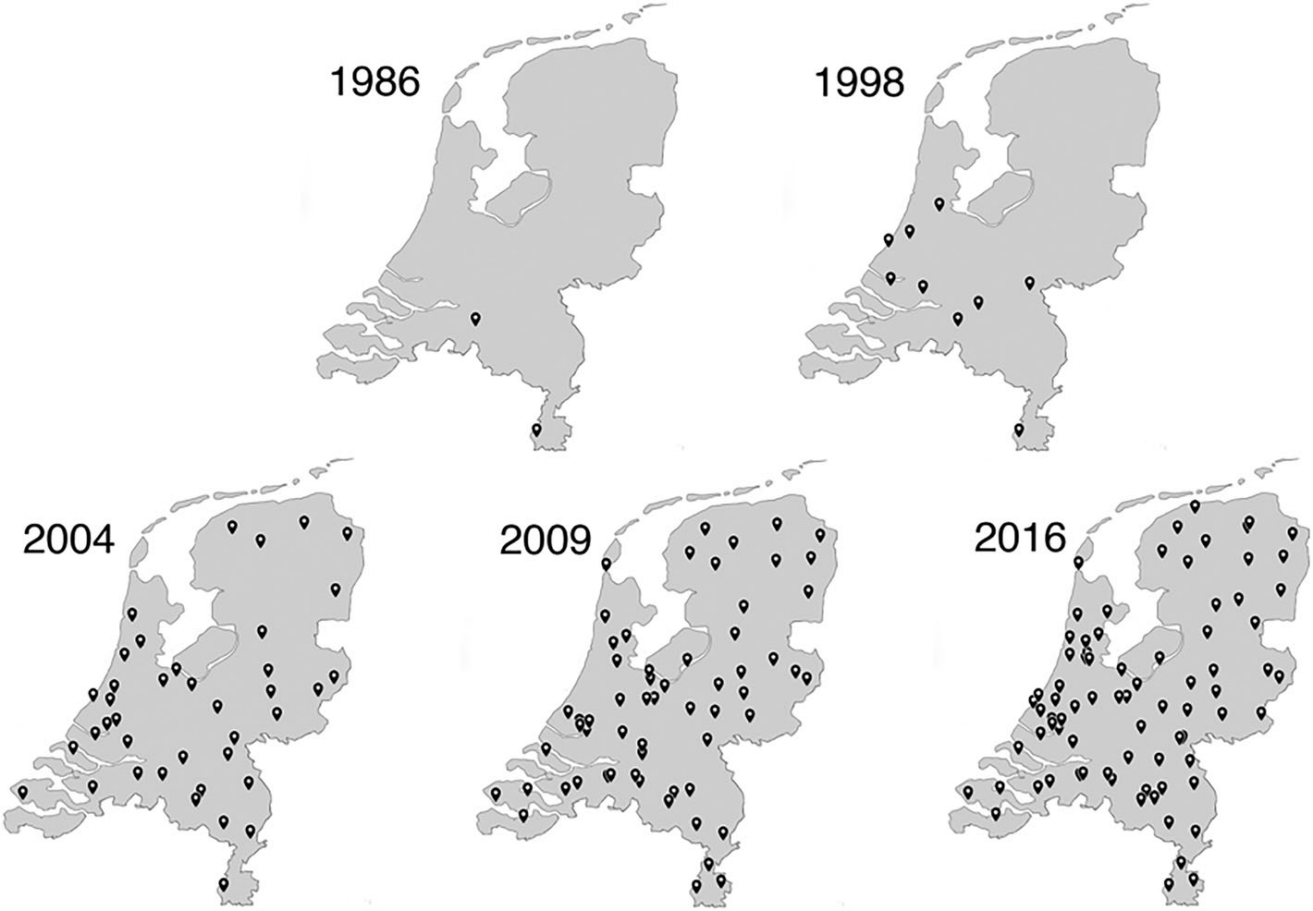
The distribution of memory clinics (MCs) in the Netherlands from 1986 to 2016

The number of MCs increased from 12 in 1998 to 43 in 2004, and from 65 in 2009 to 91 in 2016 (Figure 1). Most Dutch hospitals (71%, n = 85) had an MC. Five MCs that participated in the previous survey no longer existed.

In addition to an increase in the number and distribution of MCs, the capacity also increased over time. The average number of newly referred patients per year increased from 130 in 1998 to 199 in 2004, 225 in 2009 to 268 in 2016 (see Table 1). Since 1998, the total number of new patients increased from 1,560 to 24,388. The variation between MCs was large, with the total number of new patients ranging between 30 and 1,000 patients per center. The mean percentage of patients younger than 65 years was 20.6% ± 20.2 (range: 0%‐75%). University‐based MCs and MCs coordinated by neurology have seen more younger patients on average than non‐academic MCs (35% versus 18%, *p* = 0.02) and MCs coordinated by clinical geriatrics (35% versus 8%, *p* < .001).

**Table 1:** Development of memory clinics (MCs) from 1998 to 2016

	1998	2004	2009	2016
Number of MCs	12	43	65	91
Average no. of newly referred patients	130	199	225	268
Total no. of patients	1,560	8,557	14,625	24,388
Regional collaboration (%)	15	63	87	89
Syndrome diagnosis (%)^a^				
Dementia	85	70	59	53
Cognitive disorders, no dementia	10	24	24	25
No cognitive disorders	5	6	15	22
Number of patients with dementia	1,326	5,391	8,629	12,926
% of dementia incidence (22,200)	6	24	39	58
Diagnostics (%)				
Standardized protocol	100	78	87	97
National dementia guideline	100	50	71	82
Treatment (%)				
Pharmacological	92	97	95	91
Psychosocial interventions	38	65	53	72
Back to referral (%)	36	55	52	57

a The syndrome diagnoses were estimated averages and therefore did not always result in a mean total of 100%.

### 3.2 Organization

**Figure 2:**
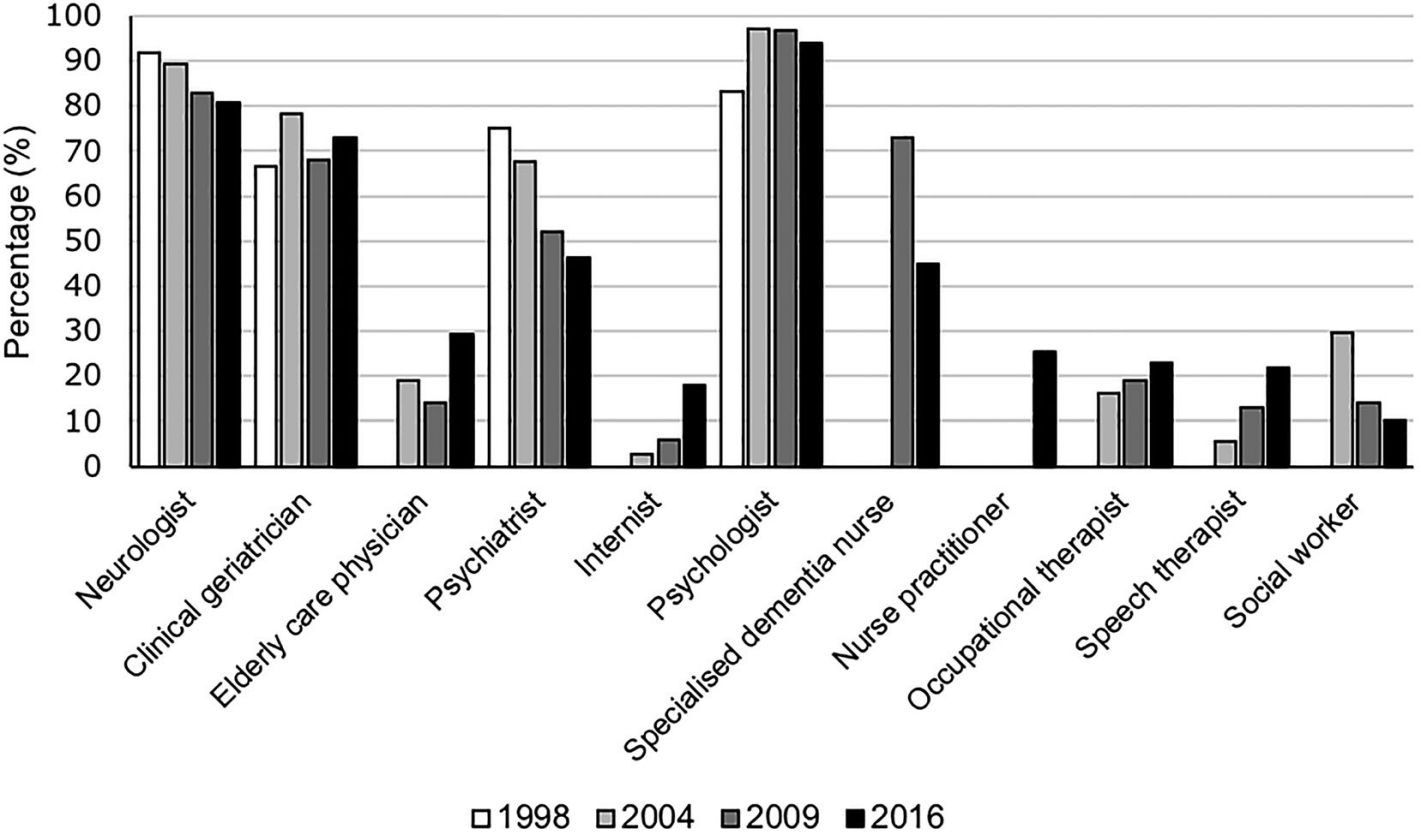
Changes in the professions involved in memory clinics (MCs) over time

In 2016, MCs were coordinated by clinical geriatricians (39%), neurologists (26%), elderly care physicians (9%), psychiatrists (1%), or (neuro)psychologists (1%). In addition, in 24% of the MCs, the coordination consisted of a collaboration between two or more disciplines. The professionals most frequently involved in MCs were neurologists (81%), clinical geriatricians (73%), psychiatrists (46%), elderly care physicians (30%), psychologists (94%), and specialized dementia nurses (45%). In comparison with 2009, fewer psychiatrists and specialized dementia nurses were involved (Figure 2). Both physician assistants and clinical nurse specialists were newly identified professionals within MCs.

In the Dutch healthcare system, doctor referral is mandatory. New patients were most often referred to MCs by a general practitioner (77%, [range: 0%‐100%]). Second opinions were obtained in 6% of the patients on average (range: 0%‐70%) and supra‐regional referrals in 5% of the patients (range: 0%‐40%). Both second opinions (22% versus 4%, *p* < .001) and supra‐regional referrals (23% versus 3%, *p* < .001) were most often carried out by university‐based MCs rather than by non‐academic MCs.

### 3.3 Diagnosis

Although dementia was still the most common syndrome diagnosis made in MCs, the proportion decreased from 85% in 1998 to 53% (range: 10%‐85%) in 2016. In contrast, the proportion of patients categorized as having “cognitive impairments without dementia” increased from 10% in 1998 to 25% (range: 10%‐50%) in 2016, and patients with “no cognitive impairments” increased from 5% in 1998 to 22% (range: 0%‐50%) in 2016 (Table 1). The mean proportion of patients without cognitive impairments was most often seen in MCs coordinated by neurologists rather than in MCs coordinated by clinical geriatricians (16% versus 7%, *p* < .001). Given the current incidence of dementia in the Netherlands of 22,200,^21^, ^22^ and the estimation that total approximately 12,926 patients were diagnosed in Dutch MCs per year, we calculated that MCs diagnosed 58% of all incident cases of dementia in 2016. This reflects an increase in the proportion of incident cases of dementia diagnosed by MCs compared with that reported in our previous surveys (Table 1, 1998: 6%; 2004: 24%; and 2009: 39%).

The most common causes of dementia were Alzheimer's disease (AD) (46%, [range: 5%‐80%]), vascular dementia (VaD) (16%, [range: 2%‐40%]), and mixed causes (20%. [range: 0%‐80%]). In addition, rarer causes of dementia were also observed, such as frontotemporal dementia (FTD) (5%, [range: 0%‐21%]), Parkinson's dementia (3%, [range: 0%‐10%]), and Lewy body dementia (6%, [range: 0%‐20%]).

### 3.4 Diagnostic assessment

In 2016, the Dutch multidisciplinary guideline Diagnostics and Treatment of Dementia^20^ was used by 82% of the MCs. The multidisciplinary guideline includes multiple diagnostic criteria, such as McKahnn.^23^ In addition, the following criteria were used: DSM‐IV‐TR (19%),^24^ DSM‐5 (9%),^25^ ICD‐10 (12%),^26^ McKahnn criteria (30%),^23^ DuBois criteria (10%),^27^ NINDS‐AIREN (45%),^28^ and Manchester Lund criteria (19%).^29^ Five MCs (6%) did not use any specific criteria.

### 3.5 Cognitive screening

In 2016, most MCs used a cognitive screening test during their intake (86%). The MMSE^30^ was most frequently used (91%). The outcome of this test was most often used to determine additional diagnostic assessments (78%) or to determine the treatment plan (52%).

### 3.6 Neuropsychological assessment

The use of an NPA increased from 50% in 1998 to 95% in 2016. The proportion of patients in whom an NPA was performed differed largely between MCs (range: 5%‐100%). The reasons for carrying out an NPA were: to support the diagnosis (92%), collect differential diagnostic information (91%), to gain insight into strengths and weakness (44%), and as a starting point for neuropsychological treatment (31%). The most often reported reason for not carrying out an NPA was if a patient had a clinically evident diagnosis of dementia, in which case a neuropsychological assessment would not have any additional diagnostic value (78%). Other reasons were lack of financial means (6%) or no possibilities within the team (1%). The cognitive tests varied per center. Nearly all MCs (96%) held multidisciplinary meetings. In 92% of the MCs, a psychologist participated in this meeting. The following topics were discussed by the psychologists: conclusion of the NPA (99%), differential diagnosis (92%), cognitive profile (85%), and advice on how to cope with cognitive complaints in daily life (79%). During the diagnostic disclosure performed by the medical doctor, the following aspects of an NPA were discussed: NPA conclusion (73%), advice on how to cope with cognitive complaints (58%), and results per cognitive domain (49%).

### 3.7 Additional assessment tools

**Figure 3:**
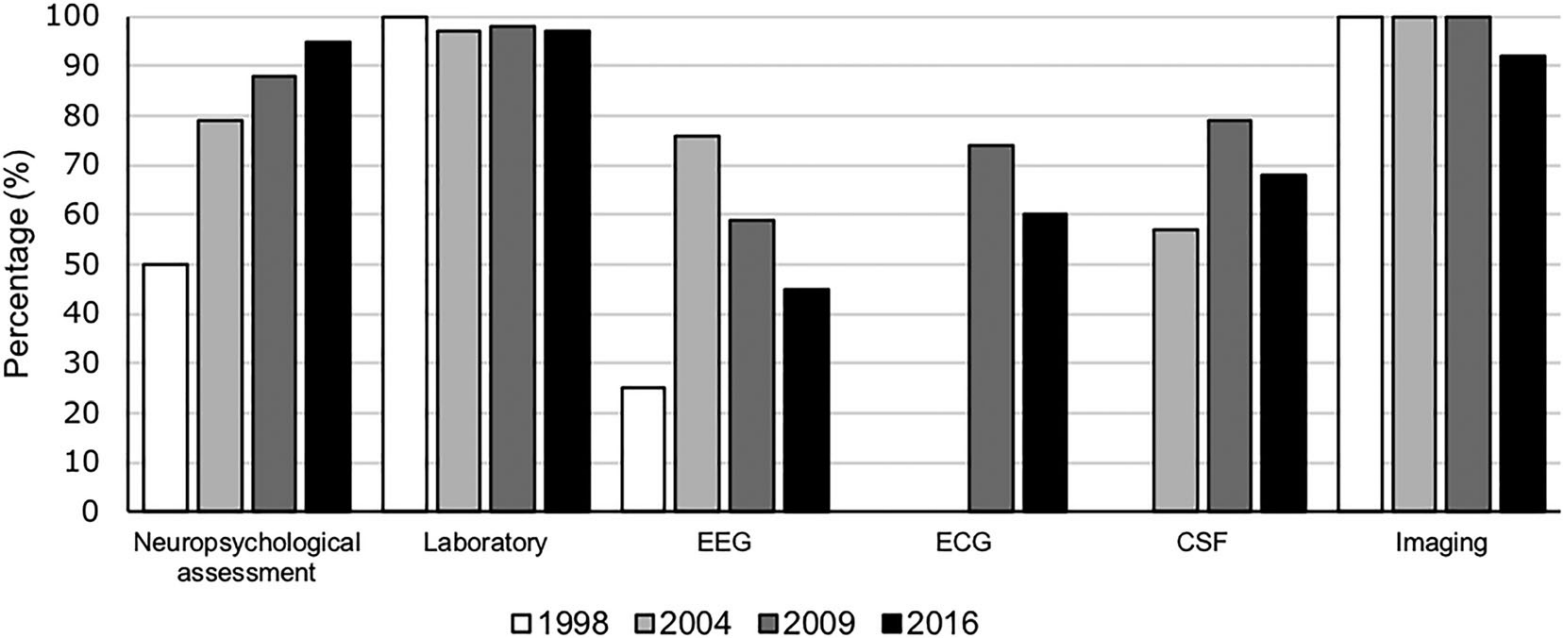
The use of various assessment tools in memory clinics (MCs) in the Netherlands over time

In addition to an NPA, laboratory tests (97%) or neuroimaging studies (92%) were frequently used in MCs. Compared with 2009, the number of MCs using neuroimaging decreased from 100% to 92%, and the number of MCs using EEG, ECG, and CSF decreased from 59% to 45%, 74% to 60%, and 79% to 68%, respectively (Figure 3). There was a large variation between MCs with respect to the assessment tools used, the percentage of patients in whom these were applied and the average estimated time (NPA: 49% [range: 5%‐100%]; 281 minutes (min) [range 63‐300 min], lab tests: 96% [range: 30%‐100%]; 9 min [range: 1‐25 min, EEG: 17% [range: 0.10%‐100%]; 47 min [range: 30‐120 min], ECG: 68% [range: 1%‐100%]; ECG: 9 min [range: 5‐15 min], CSF: 12% [range: 0.10%‐75%]; CSF: 29 min [range: 5‐60 min], neuroimaging: 82% [range: 20%‐100%]); neuroimaging: 29 min [range: 10‐60 min]).

### 3.8 Scales for behavioral and daily functioning

With regard to assessment scales, both the Geriatric Depression Scale (88%)^31^ and the Neuropsychiatric Inventory (71%)^32^ were frequently used. To measure functioning in daily living, the Lawton and Brody Instrumental Activities of Daily Living Scale (66%)^33^ and the Interview for Deterioration in Daily Living Activities in Dementia (39%)^34^ were most often used. There was a variation between MCs in the use of specific instruments for evaluating neuropsychiatric symptoms and daily living.

### 3.9 Treatment

From 1998 to 2016, pharmacological treatments were offered in approximately 90% of the MCs (Table 1). Psychosocial interventions were routinely offered in 38% of the MCs in 1998, and this increased to 72% MCs in 2016. On average 58% of the patients were referred back to the referring individual or center (10%‐100%), while 42% remained in the care of the MC (5%‐90%). For people with an MCI diagnosis, this often consisted of a follow‐up cognitive assessment after one year, and for people with a dementia diagnosis, it was a medication follow‐up.

### 3.10 Regional collaboration

Collaboration with regional healthcare organizations increased from 15% in 1998 to 89% in 2016. In 2016, 78% of all MCs were involved in regional care chains (Table 1). MCs collaborated most often with primary care (65%), mental health care (59%), and local care organizations (58%). A smaller number of MCs collaborated with other regional hospitals (28%). Structural meetings with mental health care (46%) or other local care organizations (41%) occurred in approximately half of the MCs.

## 4 DISCUSSION

This study provides new insights into the development of MCs between 1998 and 2016. The main findings showed that MCs have increased both in number and capacity and are geographically more widely distributed. In addition, our results show that MCs are better integrated within regional care facilities, and more frequent collaborations between medical disciplines have been established to coordinate MCs. The proportion of dementia diagnoses has decreased, while the proportion of milder cognitive problems has increased. Furthermore, a large variation in capacity, working methods, and in the use of additional assessments between MCs were identified.

The number of MCs has increased eightfold, the total number of newly referred patients 16‐fold, and the capacity per MC has doubled since 1998. The continuous growth in the number of MCs is in line with other countries. For example, the number of MCs increased on the British Isles from 20 in 1993 to 58 in 2000, and in Australia from 23 in 2009 to 30 in 2012.^14^, ^15^, ^16^ An English audit showed that memory services increased from 214 in 2013 to 222 in 2014.^35^, ^36^ Initially, this increase may have been partly related to the launch of anti‐dementia drugs, such as rivastigmine in the Netherlands. The further increase found in our survey might be explained by the rising prevalence and increased awareness of dementia, and the relevance of a multidisciplinary approach in the timely diagnosis of dementia.^37^ A multidisciplinary approach has been shown to have added value in differentiating between dementia subtypes, detecting comorbidity,^37^ being cost‐effective, and improving quality of life.^38^ The strong increase in patient numbers might also reflect the excellent availability of care facilities and demographic changes in the Netherlands. While the overall number of MCs has increased, five MCs have closed for the first time since our surveys began. The reasons for this are not clear. One MC reported that this was the result of a complex financial agreement between disciplines. One MC merged with another hospital. Another more speculative explanation could be that the substantial increase in MCs reached a sufficient capacity for the current demand.

MCs are by definition multidisciplinary and neurology, clinical geriatrics, and (neuro) psychology were the disciplines that were most frequently part of the MC team. Neurology and clinical geriatrics were most often involved as the leading disciplines. These numbers are comparable with what they have been over the last 20 years. Compared with previous surveys, currently, psychiatrists seem to be less involved. However, in other countries, psychiatrists are more frequently present in MCs (eg, 70%‐80% in the United Kingdom), and neurologists and geriatricians are less frequently involved.^14^, ^15^, ^39^, ^40^ The number of psychologists employed in MCs is similar to that of other countries, except for New Zealand, where only 14% of the MCs had a psychologist.^40^ These cross‐national differences may be related to historical disciplinary developments.

NPA, lab tests, and brain imaging are the most frequently used diagnostic assessment tools in MCs. This is comparable with MCs in other countries.^14^, ^16^ Since 2009, the use of an NPA has further increased, while EEG, ECG, CSF, and brain imaging tools are used by fewer MCs than in 2009 (a change of 8%‐15%). This decrease might be related to the new Dutch multidisciplinary diagnostic guideline in which CSF, for example, is not recommended as a standard routine, and neuroimaging is recommended when the etiologic cause is uncertain.^20^ Other speculative reasons might be that new MCs are smaller. The proportion of patients in whom these tools were used, however, did not change (CSF, 2009: 12% and 2016: 15%). The increase in the use of an NPA could be related to the beneficial effect of an NPA on patient outcomes (eg, accuracy of diagnosis).^41^, ^42^ Although dementia is still the most common syndrome diagnosis in MCs, diagnoses have shifted towards milder cognitive problems. This is in line with the results from the national English Audit.^35^, ^36^ This finding and the increased number of newly referred patients to MCs might be explained by the increased awareness of and attention directed towards dementia and early diagnosis in our society. The proportion of incident cases of dementia diagnosed at an MC has increased 10‐fold since 1998 from 6% to 58% (approximately 13,000 patients). Furthermore, the proportion of patients with cognitive impairment without dementia increased from 10% to 25% (approximately 6,000 patients). This is in line with the global dementia action plan, which stated that by 2025, 50% of the countries should have diagnosed at least 50% of the incident cases of dementia.^43^ The timely diagnosis of dementia is not limited to MCs but is also practiced by general practitioners (GPs) or in community mental health institutions. In the previous survey in 2009, mental health institutions were included and appeared to differ greatly from hospital‐based settings (eg, fewer disciplines and diagnostic tools available). They also often did not identify themselves as an MC. Therefore, we have focused on the development of hospital‐based MCs in this survey. A timely diagnosis of dementia is being promoted worldwide.^44^, ^45^, ^46^ The lack of a disease‐modifying treatment calls for a careful consideration of the benefits and disadvantages of an early diagnosis. Previous authors have shown that the majority of patients prefer a timely diagnosis.^47^, ^48^ Furthermore, MCs can still offer much to patients in the predementia phase, including an evaluation of the prognosis, careful monitoring of cognitive decline, and psychosocial interventions. Psychosocial interventions are now more often a part of the regular care in Dutch MCs, which is in line with the MC quality indicators.^49^

MCs started as experimental university‐based facilities. The increasing proportion of incident cases of dementia show that MCs are now accepted as mainstream healthcare facilities and as part of the standard care for the timely diagnosis of dementia. In addition, MCs offer a wider range of care and treatment options as a result of the integration with long‐term care. MCs have been criticized in the past for being unclear about whether they were running projects or contributing to regional health services.^50^ Currently, MCs are no longer isolated facilities that focus solely on conducting research. In other countries, tensions have been reported with regard to the collaboration between care services and MCs.^15^ However, in this review, we found an increased integration of MCs in long‐term care and an increased use of chain of care. Therefore, these tensions might not be present in MCs in the Netherlands. This was not explicitly asked in the present survey. The increase in regional collaborations is in line with the collaborative‐care model between primary and specialist healthcare in the Netherlands. Collaborative care, when compared with care as usual, has been shown to lead to improved outcomes (e.g., beneficial cost‐benefit ratio and health‐related quality of life).^37^, ^51^, ^52^

A large heterogeneity exists between MCs concerning the number of newly referred patients, staff members, distribution of syndrome diagnosis, use of additional assessments, cognitive instruments, and assessment scales. This heterogeneity might have different explanations. First, it might be related to differences in what an MC can offer (eg, different types of professionals within the team and financial reimbursement). Second, diversity could also be caused by differences in education and/or knowledge within the team. Third, variation in patient groups could lead to different patient‐specific needs. Harmonization of best practices could improve the collaboration between MCs, and more importantly, would make it easier to communicate and compare test results. An example of harmonization is the development of a Dutch monodisciplinary guideline for NPA in MCI and dementia, which will be available via the Dutch Institute for Psychologists (NIP). Transparency of offered MC services would enable patients to visit clinics where they would benefit from expertise related to the nature of their individual condition. To a certain extent, best practice between MCs should be shared to improve quality of care. Criticisms of MCs have also been described, such as their role in promoting stigma and issues surrounding over‐assessment of patients.^50^ In contrast, a European study has shown that MCs facilitate early referrals and to some degree battle against stigma.^53^

A strength of this current study is the repeated measurement of a comparable survey over a 20‐year period. A high response rate was obtained, and we consequently argue that the study is an adequate representation of the current situation of Dutch MCs. Nonetheless, a few drawbacks should be mentioned. Although we made utmost efforts to include every MC in the Netherlands, we may have missed some newly established MCs. In addition, we did not obtain a response from all identified MCs. The estimated numbers should therefore be carefully interpreted. The nonresponding MCs were all non‐academic hospitals, but differed in geographical location, size, and coordinating discipline. Another important point is that the results are mainly based on self‐reported estimates rather than objective data from registries.

## 5 CONCLUSION

Since 1998, MCs in the Netherlands have shown substantial development in number, geographical distribution, and total capacity. MCs are no longer isolated, university‐based facilities with a strong focus on scientific research. They are now part of the regular care for the timely diagnosis of dementia and milder cognitive disorders and are integrated into regional care chains. Among MCs, a large diversity in specific working methods and diagnostic tools was identified. This diversity should be the focus of future research to increase transparency of the working methods of individual clinics and to harmonize best practices, which will both improve quality of care in Dutch MCs.

## CONFLICT OF INTEREST

None declared.

## DATA AVAILABILITY STATEMENT

The data that support the findings of this study are available on request from the corresponding author. The data are not publicly available because of privacy or ethical restrictions.
